# An open-label, single-arm, multi-center, phase II clinical trial of single-dose [^131^I]meta-iodobenzylguanidine therapy for patients with refractory pheochromocytoma and paraganglioma

**DOI:** 10.1007/s12149-021-01699-0

**Published:** 2021-12-06

**Authors:** Anri Inaki, Tohru Shiga, Yoshito Tsushima, Megumi Jinguji, Hiroshi Wakabayashi, Daiki Kayano, Norihito Akatani, Takafumi Yamase, Yuji Kunita, Satoru Watanabe, Tomo Hiromasa, Hiroshi Mori, Kenji Hirata, Shiro Watanabe, Tetsuya Higuchi, Hiroyasu Tomonaga, Seigo Kinuya

**Affiliations:** 1 Department of Nuclear Medicine, Kanazawa University Hospital, 13-1 Takara-machi, Kanazawa, Ishikawa 920-8641 Japan; 2 Department of Clinical Research and Trial, Advanced Clinical Research Center, Fukushima Global Medical Science Center, Fukushima Medical University, 1-banchi Hikarigaoka, Fukushima, Fukushima 960-1295 Japan; 3 Department of Diagnostic Radiology and Nuclear Medicine, Gunma University Graduate School of Medicine, 3-39-15 Showa-machi, Maebashi, Gunma 371-8511 Japan; 4 Department of Radiology, Kagoshima University Graduate School of Medical and Dental Sciences, 8-35-1 Sakuragaoka, Kagoshima City, Kagoshima 890-8544 Japan; 5 Department of Functional Imaging and Artificial Intelligence, Kanazawa University, 13-1 Takara-machi, Kanazawa, Ishikawa 920-8641 Japan; 6 Department of Diagnostic Imaging, Hokkaido University Graduate School of Medicine, 5-chome Kita-14-jou, Kita-ku, Sapporo, Hokkaido 060-8648 Japan

**Keywords:** Pheochromocytoma and paragangliomas, [^131^I]meta-iodobenzylguanidine, Phase II study

## Abstract

**Objective:**

In this phase II study, we aimed to investigate the efficacy and safety of single-dose [^131^I]meta-iodobenzylguanidine (^131^I-mIBG) therapy in patients with refractory pheochromocytoma and paraganglioma (PPGL).

**Patients and methods:**

This study was designed as an open-label, single-arm, multi-center, phase II clinical trial. The enrolled patients were administered 7.4 GBq of ^131^I-mIBG. Its efficacy was evaluated 12 and 24 weeks later, and its safety was monitored continuously until the end of the study. We evaluated the biochemical response rate as the primary endpoint using the one-sided exact binomial test based on the null hypothesis (≤ 5%).

**Results:**

Seventeen patients were enrolled in this study, of which 16 were treated. The biochemical response rate (≥ 50% decrease in urinary catecholamines) was 23.5% (90% confidence interval: 8.5–46.1%, *p* = 0.009). The radiographic response rates, determined with CT/MRI according to the response evaluation criteria in solid tumors (RECIST) version 1.1 and ^123^I-mIBG scintigraphy were 5.9% (0.3%–25.0%) and 29.4% (12.4%–52.2%), respectively. The most frequent non-hematologic treatment-emergent adverse events (TEAEs) were gastrointestinal symptoms including nausea, appetite loss, and constipation, which were, together, observed in 15 of 16 patients. Hematologic TEAEs up to grade 3 were observed in 14 of 16 patients. No grade 4 or higher TEAEs were observed. All patients had experienced at least one TEAE, but no fatal or irreversible TEAEs were observed.

**Conclusion:**

A single dose ^131^I-mIBG therapy was well tolerated by patients with PPGL, and statistically significantly reduced catecholamine levels compared to the threshold response rate, which may lead to an improved prognosis for these patients.

## Introduction

Pheochromocytomas and paragangliomas (PPGLs) are rare tumors, genealogically derived from the neural crest cells that develop into the sympathetic and parasympathetic nervous systems. The World Health Organization (WHO) classification defines pheochromocytomas as tumors arising from the adrenal medulla, and paragangliomas as those arising from extra-adrenal chromaffin tissue [1]. The incidence of PPGL is 2 to 8 per million individuals per year and approximately 90% of all PPGLs are resectable at initial diagnosis; but 10% to 30% are locally unresectable or metastatic [2–4]. Although the 5-year survival rate of resectable PPGL is estimated at approximately 90%, that of unresectable or metastatic PPGL is reduced to 40–50%, which is why the latter is considered refractory PPGL [5]. Although there is no standardized treatment strategy for refractory PPGL, systemic treatments such as chemotherapy and radiation therapy are currently recommended [6].

[^131^I]meta-iodobenzylguanidine (^131^I-mIBG) is a radioactive agent with high-energy beta-ray emission, first developed by Wieland et al. in 1980 [7]. It is specifically taken up through neuronal uptake-1 transporter into tumor cells derived from the abovementioned neural crest cells, such as pheochromocytomas, medullary thyroid cancer, and neuroblastoma, and its high-energy, cytotoxic, beta-ray emission has an anticancer effect on these tumors. Several studies revealed that the response rate of these tumors to ^131^I-mIBG therapy, as determined with imaging and in hormonal examination, was 0–83% and 20–100%, respectively [8–11]. However, there have been only few prospective studies due to the extremely low incidence of PPGL [12]. Based on those background, in December 2012, the Advanced Medical Care Committee, sponsored by the Japanese Ministry of Health, Labour and Welfare, promoted ^131^I-mIBG to the status of an anticancer drug with high priority for clinical development. Therefore, we performed ^131^I-mIBG therapy in the Japanese Advanced Medical Care Program B and reported its safety and efficacy as primary and secondary outcomes, respectively [12, 13]. Based on those results, we conducted this phase II clinical trial to investigate the efficacy of single-dose ^131^I-mIBG therapy in patients with refractory PPGL. This study was designed and data provided by FUJIFILM Toyama Chemical Co., Ltd.

## Patients and methods

### Study outline and ethical considerations

**Fig. 1:**
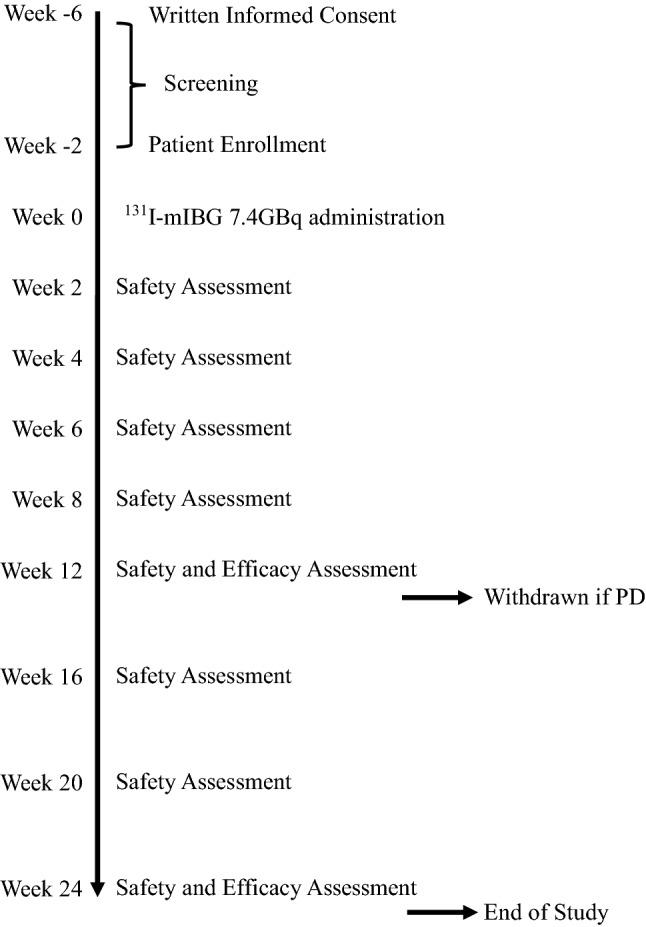
Study protocol. Physical and blood examinations were performed in each safety assessment and urinary catecholamine, CT, ^123^I-mIBG scintigraphy and QOL questionnaire in each efficacy assessment

This study was designed as an open-label, single-arm, multi-center, phase II clinical trial. Figure 1 is a flow diagram of the visits of the study. After providing written informed consent, the enrolled patients received a fixed dose of ^131^I-mIBG. Its efficacy was evaluated 12 and 24 weeks after ^131^I-mIBG administration, and its safety was evaluated continuously during the follow-up period until the end of the study (24 weeks after ^131^I-mIBG administration). The primary endpoint was defined as the biochemical response in terms of urinary catecholamines (adrenaline, noradrenaline, metanephrine, and normetanephrine) collected for 24 h. Secondary endpoints were defined as the objective response rate (ORR) upon CT or MRI according to the response evaluation criteria in solid tumors (RECIST) version 1.1; the scintigraphic response upon ^123^I-mIBG scintigraphy; patients’ quality of life (QOL) according to the European Organization for Research and Treatment of Cancer (EORTC) QLQ-C30, EuroQol 5 Dimensions (EQ-5D-5L) questionnaire; and patients’ safety [14, 15].

This study was approved by the institutional review board of each involved institution, and was preregistered in a public database (JapicCTI-173751) [16]. All operational notifications and procedures required for this study were properly implemented based on the rules and principles of the 1964 Declaration of Helsinki, the Japanese Ministerial Ordinance on Good Clinical Practice, and other related laws and regulations.

### Patients

Patients with confirmed refractory PPGL were enrolled into this study. Refractory PPGLs were defined as locally progressive or metastatic PPGLs that were unresectable and not amenable to curative radiation therapy at their initial diagnosis or at recurrence.

Inclusion criteria were as follows: the presence of a measurable lesion according to RECIST version 1.1; ^123^I-mIBG avidity of at least one measurable lesion; a urinary catecholamine value more than three times the standard value; adequate bone marrow, renal, hepatic, cardiac, and respiratory function; absence of uncontrolled diabetes mellitus; an Eastern Cooperative Oncology Group (ECOG) performance status of 0–1; a life expectancy ≥ 6 months; independent feeding, excretion, and sleeping; age ≥ 20 years; and provision of written informed consent.

Exclusion criteria were as follows: having other malignancies except for thyroid medullary carcinoma with multiple endocrine neoplasia type 2, hemangioblastoma of the retina with von Hippel-Lindau disease, and neurofibroma with neurofibromatosis type 1; a history of ^131^I-mIBG therapy; taking any drugs interfering with ^131^I-mIBG uptake; alpha-methyl-L-tyrosine (α-MPT) dependency; having underwent surgical operation, chemotherapy, or transcatheter arterial embolization of liver metastasis in the previous 8 weeks; untreated or insufficiently treated Common Terminology Criteria for Adverse Events (CTCAE) grade ≥ 2 non-hematologic toxicity due to previous treatment; active infection of hepatitis B virus, hepatitis C virus, human immunodeficiency virus, or other severe infections; immunodeficiency; a history of uncontrollable increase of catecholamines; a history of fatal arrhythmia or asystole; uncontrollable symptomatic arrhythmia, thyroid dysfunction, respiratory disease, or pleural effusion and ascites; coronary artery disease, amiodarone-dependent arrhythmia, severe valvular disease, aortic disease, and/or a bleeding disorder; pregnant or lactating women; women who were planning to become pregnant; psychosis; not suitable for isolation for radiation control; a history of allergic reactions to potassium iodide; and/or a need for palliative radiation therapy.

### Treatment

^131^I-mIBG (FUJIFILM Toyama Chemical Co., Ltd., Tokyo, Japan) was intravenously administered as a single dose of 7.4 GBq over 1 h. In institutions where the legally permitted dose of ^131^I-mIBG was lower than 7.4 GBq, patients received 5.5 GBq. As essential concomitant agents, 300 mg/day of potassium iodide was administered from at least 24 h before to 7 days after ^131^I-mIBG administration, and antiemetics (5-HT3 receptor antagonist) were prescribed before ^131^I-mIBG administration, to prevent radiation-induced hypothyroidism and gastrointestinal symptoms, respectively. The patients were treated in the radiation treatment room and discharged from the radiation treatment room when they satisfied the release criteria as determined by the applicable Japanese regulations. All treatments, including chemotherapy, radiation therapy, interventional radiology, and α-MPT therapy were prohibited during treatment and the follow-up period.

### Efficacy evaluation

Urinary catecholamine levels were measured and radiological examinations (CT or MRI), ^123^I-mIBG scintigraphy, and QOL questionnaires were conducted 12 weeks and 24 weeks after ^131^I-mIBG administration and compared with those determined and conducted at the time of screening.

The biochemical response in terms of urinary catecholamines was graded into the following four categories according to previous reports based on the WHO classification [10, 17, 18]:

- Complete response (CR): all urinary catecholamine levels were within normal range;
- Partial response (PR): any urinary catecholamine levels were reduced by 50% or more;
- Progressive disease (PD): any urinary catecholamine levels increased by 25% or more;
- Stable disease (SD): none of the above.

The objective response was assessed by using CT or MRI, according to the RECIST version 1.1, by an independent data review board [19]. We also evaluated the scintigraphic response according to the semi-quantitative method previously reported by our study groups [12]. In short, the scintigraphic response was classified into CR, PR, PD, and SD according to the reduction rate of ^123^I-mIBG accumulation at 12 and 24 weeks after ^131^I-mIBG administration compared to the baseline in each lesion. The change from the baseline in patients’ QOL was evaluated using EORTC QLQ-C30 and EQ-5D-5L.

### Safety evaluation

Symptoms and physical findings, including blood pressure, pulse rate, body temperature, and oxygen saturation, were evaluated every hour until 5 h after ^131^I-mIBG administration to detect acute toxicity of ^131^I-mIBG. Symptoms, physical findings, and blood examinations were also evaluated every 2 weeks until 8 weeks after administration, and every 4 weeks from 12 to 24 weeks after administration, to detect medium- to long-term hematologic and non-hematologic toxicity.

Types and degrees of adverse events were described according to the CTCAE version 4.0, as translated into Japanese by the Japan Clinical Oncology Group (CTCAE v4.0—JCOG) [20]. Adverse events that occurred after ^131^I-mIBG administration were defined as treatment-emergent adverse events (TEAEs) and were included in the statistical analysis. The relationship between ^131^I-mIBG administration and each TEAE was assessed by the investigators at each institution.

### Statistical analysis

The full analysis set (FAS) consisted of all patients who enrolled in this study, and efficacy analyses were performed based on FAS. The primary endpoint was the biochemical response rate in terms of urinary catecholamines, defined as the proportion of patients with CR and PR. We calculated the biochemical response rate and its two-sided Clopper–Pearson’s 90% confidence interval (CI). Furthermore, the one-sided exact binomial test was used to evaluate the biochemical response rate based on the null hypothesis (≤ 5%). The secondary endpoints (the ORR determined with CT or MRI, the scintigraphic response rate, and their two-sided 90% CIs) were calculated in the same way as the primary endpoint.

According to a previous report, we calculated the minimum required sample size based on the exact binominal test with a one-sided type-I error of 0.05 and a two-sided type-II error of 0.80 [10]. The sample size was fixed at 13.

Continuous variables are presented as means and standard deviations as appropriate, and categorical variables are presented as counts. A *p* value of less than one-sided 5% was defined as a statistically significant difference. Safety was assessed in all the patients who received the study drug. Statistical analyses were performed with SAS software, version 9.4 (SAS Institute).

## Results

### Patients

From January 2018 to January 2020, 17 patients were enrolled in this study. The demographic information of all patients is summarized in Table 1. An elevation of the urinary normetanephrine level was observed in all patients. Three patients (18%) had undergone chemotherapy and three patients (18%) had undergone radiation therapy before ^131^I-mIBG therapy. Eleven patients (65%) had bone metastasis at enrollment. One patient withdrew the trial before receiving the protocol treatment. The target administration dose was set at 7.4 GBq in 14 patients and 5.55 GBq in 2 patients, the latter being the maximum permissible dose of the respective institution. The mean ± standard deviation administered dose was 7.24 ± 0.88 GBq (range, 5.09–8.69 GBq). Follow-up was discontinued in two patients (13%) because of disease progression at the 12-week follow-up and in one patient (6%) because of a serious adverse event (admission due to disease progression) at 20 weeks after ^131^I-mIBG therapy. Thus, the number of patients who completed the 24-week follow-up was 13.

**Table 1:** Patient demography in full analysis set

Characteristic	(*N* = 17)
Age (years)^a^	59.1 ± 15.2
Sex (Female/Male)	
Female	10
Male	7
Weight (kg) ^a^	58.1 ± 10.3
BMI^a^	22.0 ± 2.8
ECOG performance status	
0	14
1	3
Diagnosis	
Pheochromocytoma	13
Paraganglioma	4
Definition of refractory PPGL	
Locally progressive at initial diagnosis	0
Metastatic at initial diagnosis	2
local recurrence	1
Recurrence of metastasis	14
Previous treatment (duplicated)	
Surgical operation	16
Chemotherapy	3
Radiotherapy	3
Others	5
Elevation of urinary catecholamines at enrollment (Duplicated)	
Adrenaline	1
Noradrenaline	6
Metanephrine	3
Normetanephrine	17
Organ of target lesion in RECIST criteria (Duplicated)	
Adrenal gland	1
Bone	5
Liver	3
Lung	3
Lymph node	5
Peritoneum	1
Pleura	2
Others	5
Number of successful ^131^I-mIBG administration	16
Actual administration dose (GBq)^a^	7.24 ± 0.88
Number of successful follow-up at 24 weeks after treatment	13
Number of deaths during follow-up period	1

^a^Data shown as Mean ± SD

### Biochemical response in terms of urinary catecholamines

**Fig. 2:**
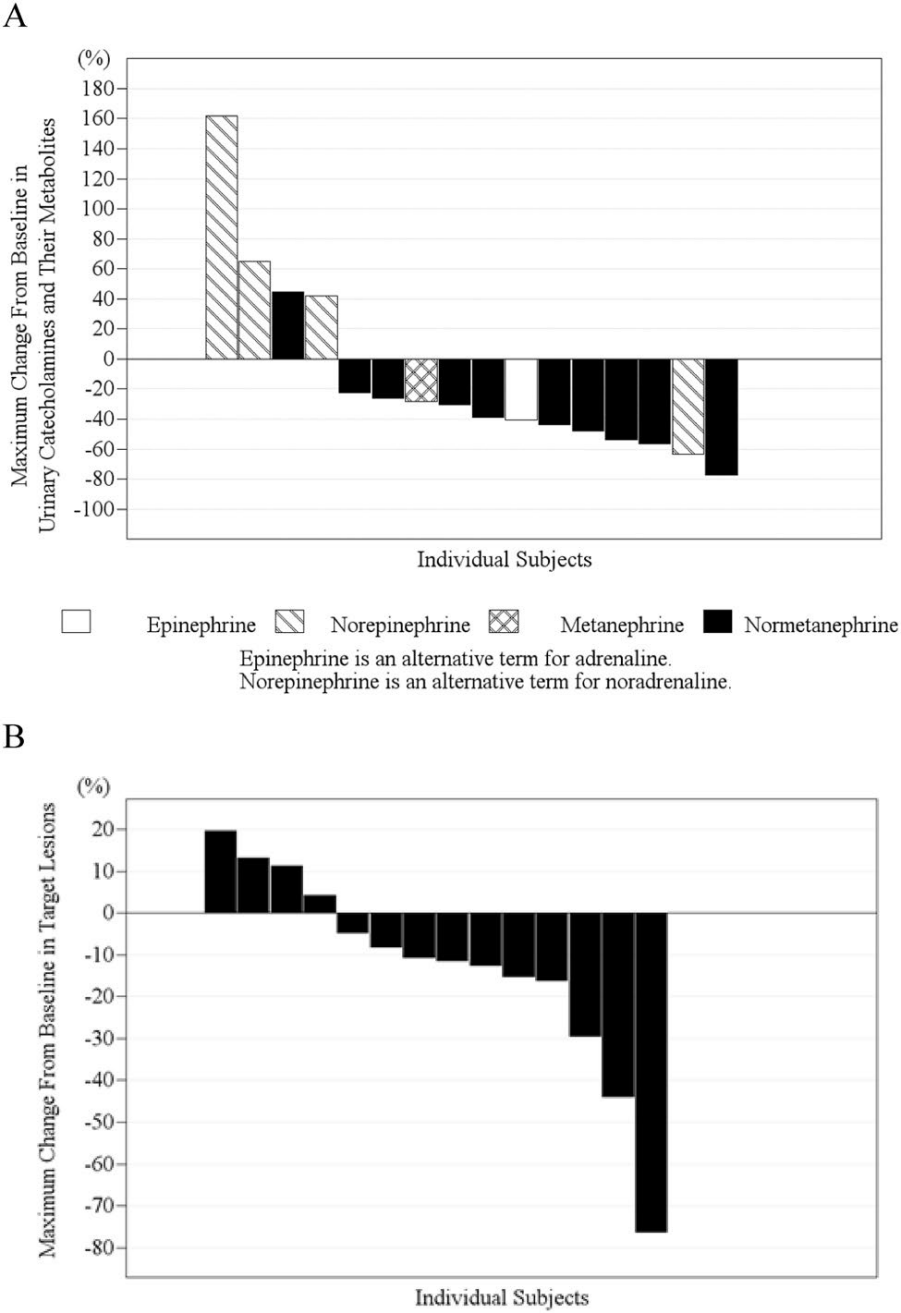
Waterfall plot of biochemical best response of urinary catecholamines (**A**) and objective response evaluated according to RECIST v1.1 criteria (**B**)

The best biochemical response in terms of urinary catecholamines is summarized in Table 2 and Fig. 2A. A decrease in urinary catecholamines was observed in 12 patients (75%). Although no CR was achieved, a biochemical response (CR + PR) was observed in four patients (response rate = 23.5%, 90% CI 8.5%–46.1%), which was significantly higher than the threshold response rate (*p* = 0.009). The biochemical response rate exhibited no statistically significant relationship to age, the presence of bone metastasis, the administration dose, and the history of previous treatment.

**Table 2:** Response of urinary catecholamines, objective response and scintigraphic response in full analysis set

	No. of patients (*N* = 17)		
	Urinary catecholamines	Objective response	Scintigraphic response
CR	0	0	0
PR	4	1	5
SD	8	12	8
(Non-CR/Non-PD)^a^	(Not defined)	2	0
PD	4	1	3
NE	1	1	1
Response Rate (90% CI)^b^	23.5% (8.5—46.1%)	5.9% (0.3—25.0%)	29.4% (12.4—52.2%)
Binomial Test^c^	*p* = 0.009	–	–

^a^Despite assessed measurable by each institute, no measurable lesion was found by the independent data review board

^b^Clopper-Pearson's exact confidence interval

^c^One-sided test (type I error = 5%) based on the null hypothesis (threshold response rate = 5%)

### Objective response

The evaluation of the objective response with CT or MRI by the independent data review board is summarized in Table 2 and Fig. 2B. Because no measurable lesions were observed in two patients who had been determined to have any measurable lesions by the investigators at each institution, these patients were diagnosed as non-CR/non-PD. Only one patient (ORR = 5.9%, 90% CI 0.3%–25.0%) exhibited PR, while a reduction in tumor size was observed in 10 patients (63%).

### Scintigraphic response

The scintigraphic response in terms of reduction in ^123^I-mIBG accumulation is also summarized in Table 2. A scintigraphic response (CR + PR) was observed in five patients (response rate = 29.4%, 90% CI 12.4–52.2%).

### Safety

**Fig. 3:**
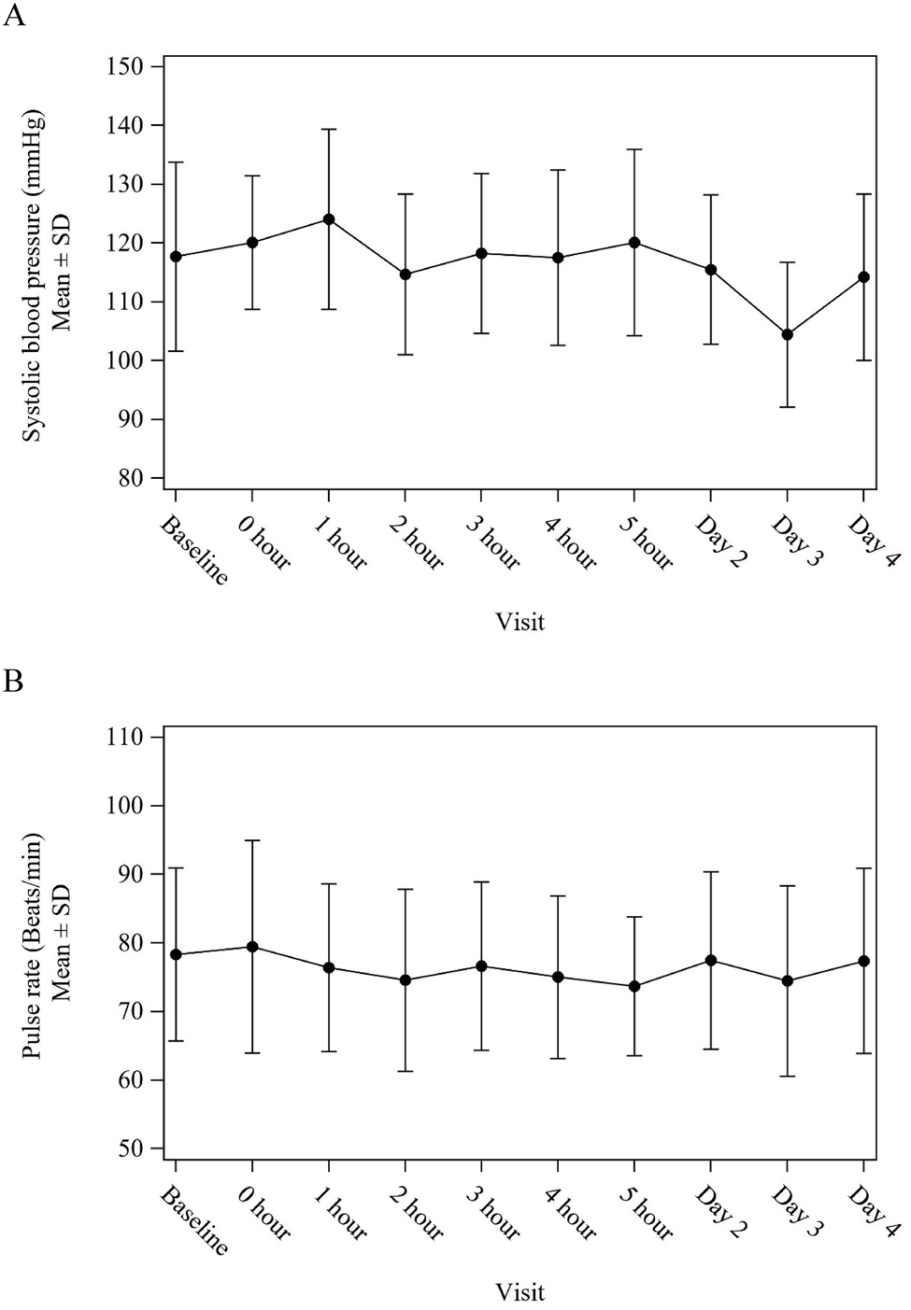
The changes of systolic blood pressure (**A**) and heart rate (**B**) during admission for ^131^I-mIBG administration

**Fig. 4:**
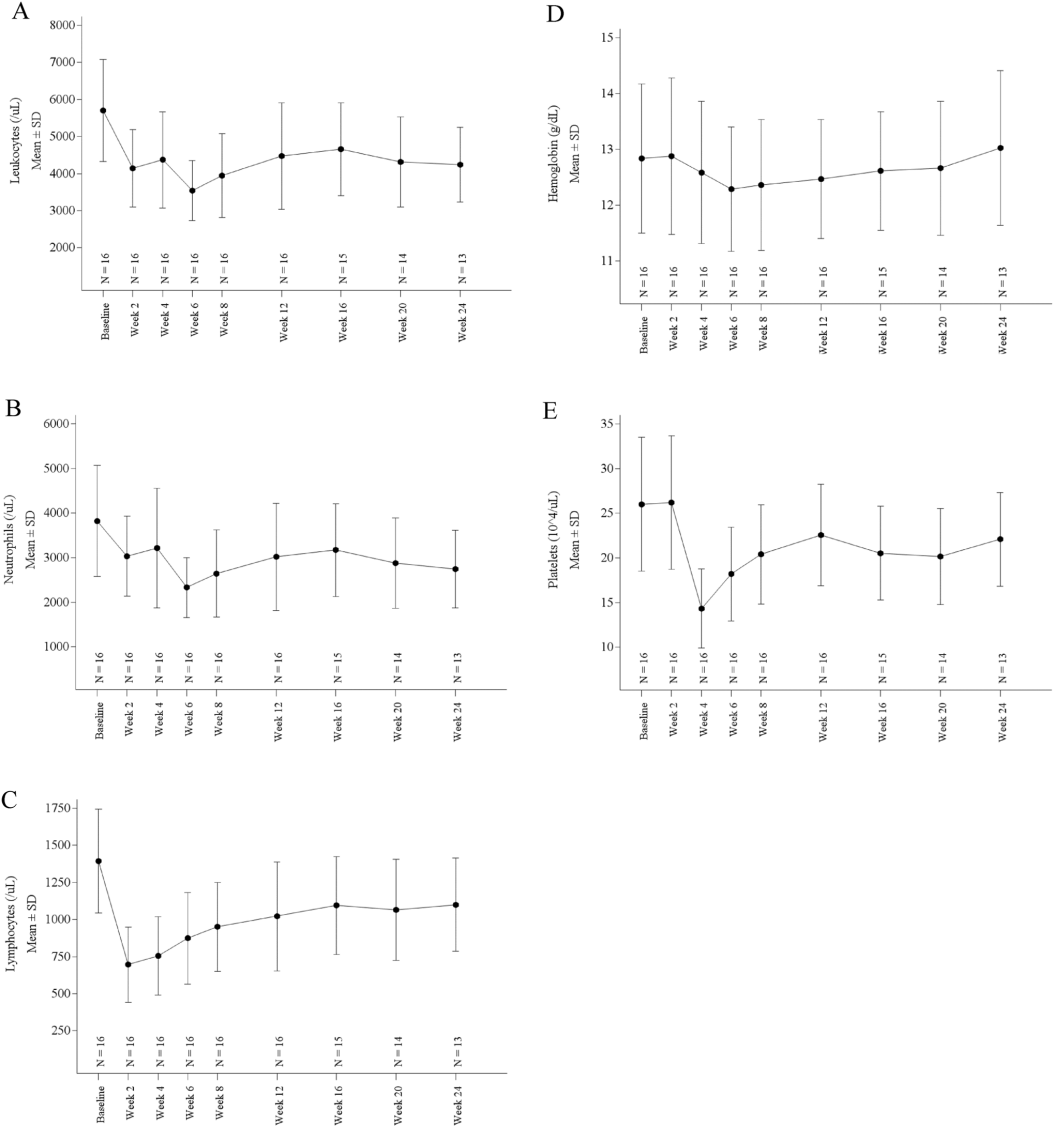
The changes of blood cells. (**A**) Leukocytes, (**B**) Neutrophils, (**C**) Lymphocytes, (**D**) Hemoglobin and (**E**) Platelets

A summary of treatment-related TEAEs is provided in Table 3. All patients experienced at least one TEAE. Notable non-hematologic TEAEs were nausea in 11 patients (69%; 2 with grade 2 and 9 with grade 1), decreased appetite in 6 (38%; 1 with grade 2 and 5 with grade 1), headaches in 6 (38%; 1 with grade 2 and 5 with grade 1), malaise in 5 (31%; all grade 1), and constipation in 7 (44%; all grade 1). Grade 3 hypertension was experienced by one patient (6%), the only non-hematologic TEAE with a grade ≥ 3. On the other hand, no statistical differences were observed in patients’ blood pressure and pulse rate at admission for ^131^I-mIBG administration compared with those at baseline (Fig. 3). Hematologic TEAEs that occurred were lymphocytopenia in 13 patients (81%; 5 with grade 3, 5 with grade 2, and 3 with grade 1), thrombocytopenia in 10 (63%; all with grade 1), leukopenia in 7 (44%; 6 with grade 2 and 1 with grade 1), and neutropenia in 4 (25%; all with grade 1). No patients experienced infectious disease due to leukopenia. The changes in leukocytes, neutrophils, lymphocytes, hemoglobin, and platelets after ^131^I-mIBG administration are presented in Fig. 4. The nadir was observed at 2 weeks after ^131^I-mIBG administration for lymphocytes, at 4 weeks for platelets, and at 6 weeks for leukocytes, neutrophils, and hemoglobin. One patient (6%) died due to progression of the primary disease.

**Table 3:** Treatment-Related Adverse Events

System organ class	No. of patients (N = 16)			
Terms	Grade 1	Grade 2	Grade 3	Grade 4
Cardiac disorders	1	1		
Palpitations		1		
Left ventricular dysfunction	1			
Gastrointestinal disorders	13	2		
Abdominal discomfort	1			
Constipation	5			
Dyspepsia	1			
Nausea	9	2		
Salivary gland pain	1			
Stomatitis	1			
Vomiting	1			
Noninfective sialadenitis	2			
General disorders and administration site conditions	5	1		
Malaise	5			
Pain		1		
Investigations	4	5	5	
Blood bilirubin increased	1			
Lymphocyte count decreased	3	5	5	
Neutrophil count decreased	4			
Platelet count decreased	10			
White blood cell count decreased	1	6		
Brain natriuretic peptide increased	2			
Metabolism and nutrition disorders	5	1		
Decreased appetite	5	1		
Musculoskeletal and connective tissue disorders	1			
Arthritis	1			
Nervous system disorders	5	1		
Headache	5	1		
Reproductive system and breast disorders	1			
Amenorrhea	1			
Vascular disease	1		1	
Hypertension	1		1	

MedDRA ver. 23.0

TEAEs from onset the study drug administration to week 24 (or discontinuation of the study) were counted

If a patient experienced more than one event, the analysis was done on the basis of most severe event

### Quality of life

In the EORTC QLQ-C30 and EQ-5D-5L questionnaires, there were no statistically significant differences between the baseline scores and the scores after 12 and 24 weeks, including in the pain and constipation scores.

## Discussion

In summary, we clarified the efficacy and safety of ^131^I-mIBG therapy in patients with PPGLs. We aimed to determine whether the therapy could reduce urinary catecholamines, as the primary endpoint of the study. This outcome was previously reported as an independent prognostic factor, with the causes of death including cardiac disorders such as fatal arrhythmia and cardiac dysfunction due to elevated catecholamine levels in patients with PPGL [21, 22]. We hypothesized that a decrease in the catecholamine level would improve patients’ prognosis and symptoms. In addition, no grade ≥ 4 hematologic or non-hematologic toxicity occurred in this study. Grade 3 hypertension was observed as the only severe adverse reaction, and this symptom improved without any sequelae. Herein, we concluded that ^131^I-mIBG therapy is well tolerated by and effective for patients with PPGL.

In this study, the response rate evaluated with CT or MRI was lower than those by scintigraphy and urinary catecholamines, which was also observed in previous studies [11, 12, 23]. We consider that this is mainly because functional or metabolic changes preceded anatomical changes in affected tumors. In addition, as tumor size base on RECIST dose not reflect total tumor volume, evaluating tumor activity in terms of scintigraphy and urinary catecholamines is considered to be more appropriate especially in patients with unmeasurable lesions such as bone metastasis.

^131^I-mIBG therapy has been attempted for the treatment of mIBG-avid, unresectable tumors such as PPGL and neuroblastoma since the development of mIBG. However, as PPGL is rare, most studies were performed on small study populations or were of a single-center or observational nature [10, 18, 24–32]. A meta-analysis was conducted in the attempt to determine the efficacy of ^131^I-mIBG therapy in a larger number of patients; however, it was subject to limitations with regard to a lack of uniform objective evaluation of adverse events in the studies being analyzed [11]. In this study, we performed ^131^I-mIBG therapy according to a standardized protocol, in which combined treatments such as extra-beam radiotherapy, chemotherapy, and α-MPT were prohibited, and the safety and efficacy were evaluated quantitatively.

As the optimal treatment strategy for malignant PPGL remains to be established, various systemic treatments have been attempted, including chemotherapy with cyclophosphamide, vincristine, and dacarbazine (CVD therapy). A meta-analysis of CVD therapy revealed that the rates of CR, PR, and SD upon imaging were 4%, 37%, and 14%, respectively [33]. On the other hand, a recent report indicated no statistically significant difference in overall survival between responders and non-responders to CVD therapy [34]. Furthermore, CVD therapy prior to ^131^I-mIBG therapy may improve the prognosis [24]. Further studies will be needed to determine the optimal order and possible interactions of CVD and ^131^I-mIBG therapies.

Nausea, appetite loss, constipation, headache, and malaise were each experienced by more than 30% of the patients in this study. Despite prophylactic administration of antiemetics, gastrointestinal symptoms were the most common complications. Although there is little evidence for the utility of antiemetics for radioisotope therapy, various guidelines recommend prophylactic administration of 5-HT3 receptor antagonists to individuals at moderate to high risk of radiation-induced nausea and vomiting [35–38]. Furthermore, long-acting antiemetic agents may be desirable because radiopharmaceuticals increase the radiation exposure time compared with conventional external beam radiation, and the former is preferable to reduce radiation exposure to caregivers. We believe that the constipation resulted mainly from the temporary elevation of catecholamines due to tumor lysis, and decreased activity due to isolation of patients in the radiation treatment room. In fact, constipation improved within few weeks after ^131^I-mIBG administration in most patients. However, as constipation due to PPGL frequently becomes severe and sometimes fatal, appropriate treatment, such as abundant ingestion of liquids, stimulant laxatives, and stool softeners, is recommended even if symptoms are mild [39]. In this study, headaches and hypertension occurred in six and two patients, respectively. Although one patient experienced both headaches and hypertension, both had developed by the 4-week follow-up. There was insufficient evidence to confirm that the ^131^I-mIBG therapy caused the headaches. Nevertheless, the headaches possibly resulted from acute hypertension due to the elevation of catecholamines; therefore, fatal hypertension should be excluded.

Several guidelines mention the efficacy of ^131^I-mIBG therapy as palliative treatment against symptoms of catecholamine secretion [40, 41]. In this study, there were no statistically significant improvements in patients’ symptoms between baseline and after ^131^I-mIBG therapy, including hypertension, tachycardia, and constipation, all characteristic of PPGL. One explanation is that most parameters of the EORTC QLQ-C30 questionnaire at baseline were within the normal range because of symptomatic treatment that had already been performed. In addition, we excluded patients with severe disease states such as α-MPT dependency, uncontrollable elevation of catecholamines, and symptomatic arrhythmia, for whom palliation of symptoms would have been expected upon ^131^I-mIBG therapy.

The WHO has recently suggested that PPGL be included in the group of neuroendocrine neoplasms, which are derived from neuroendocrine cells and synthesize and secrete various neuroendocrine hormones [6, 42]. In this framework, PPGLs are classified as low-grade (grade 1) or intermediate-grade (grade 2) neuroendocrine tumors (NETs), regardless of the presence of metastatic lesions. However, it is clear that metastatic lesions substantially reduce the survival rate; hence, treatment strategies, including mIBG therapy for unresectable PPGLs, should be further developed. On the other hand, peptide receptor radionuclide therapy (PRRT) with radiolabeled somatostatin analogs such as ^177^Lu-DOTA-(Thy^3^)-octreotate (^177^Lu-DOTA-TATE) has recently been developed for patients with NETs, and researchers have reported its efficacy against PPGL [43, 44]. Therefore, PRRT may be an alternative treatment strategy in patients with PPGL with poor ^123^I-mIBG uptake or an insufficient therapeutic effect obtained with ^131^I-mIBG.

### Limitations

Because of the small number of patients, we could not assess dose-dependent efficacy and toxicity via a dose-escalation study. In addition, the efficacy of repeated ^131^I-mIBG administration was not assessed. Moreover, the long-term prognosis of patients treated with ^131^I-mIBG, including effects such as chronic radiation injury, was not evaluated. Finally, we did not evaluate the relationship between the efficacy of ^131^I-mIBG and the presence of genetic mutations which induce the onset of PPGLs.

## Conclusion

A single dose of ^131^I-mIBG therapy statistically significantly reduced catecholamine levels compared to null hypothesis (threshold response rate ≤ 5%) in patients with PPGL, which may lead to an improved prognosis for these patients. As the observed, short-term adverse reactions of ^131^I-mIBG administration were not as severe as to discontinue the study, the patients were considered to tolerate the therapy with a sufficient safety margin at a single dose of 7.4 GBq.
